# 3-D Worm Tracker for Freely Moving *C. elegans*

**DOI:** 10.1371/journal.pone.0057484

**Published:** 2013-02-21

**Authors:** Namseop Kwon, Jaeyeon Pyo, Seung-Jae Lee, Jung Ho Je

**Affiliations:** 1 X-ray Imaging Center, Pohang University of Science and Technology, Pohang, South Korea; 2 School of Interdisciplinary Bioscience and Bioengineering, Pohang University of Science and Technology, Pohang, South Korea; 3 Department of Materials Science and Engineering, Pohang University of Science and Technology, Pohang, South Korea; 4 Department of Molecular and Life Science, Pohang University of Science and Technology, Pohang, South Korea; 5 World Class University Information Technology Convergence Engineering, Pohang University of Science and Technology, Pohang, South Korea; Harvard University, United States of America

## Abstract

The manner in which the nervous system regulates animal behaviors in natural environments is a fundamental issue in biology. To address this question, *C. elegans* has been widely used as a model animal for the analysis of various animal behaviors. Previous behavioral assays have been limited to two-dimensional (2-D) environments, confining the worm motion to a planar substrate that does not reflect three-dimensional (3-D) natural environments such as rotting fruits or soil. Here, we develop a 3-D worm tracker (3DWT) for freely moving *C. elegans* in 3-D environments, based on a stereoscopic configuration. The 3DWT provides us with a quantitative trajectory, including the position and movement direction of the worm in 3-D. The 3DWT is also capable of recording and visualizing postures of the moving worm in 3-D, which are more complex than those in 2-D. Our 3DWT affords new opportunities for understanding the nervous system function that regulates animal behaviors in natural 3-D environments.

## Introduction

The nematode *C. elegans* has been widely used as a model animal for behavioral neuroscience due to its experimental amenability, fully sequenced genome and simple nervous system. Furthermore, *C. elegans* is the only animal whose neural system has been completely mapped [1]. In general, the behavioral neuroscience of the nematode starts from an understanding of its locomotion.

The worm's behavior or motion has been investigated primarily by using single-worm or multi-worm trackers [2]–[7]. A single-worm tracker tracks each worm precisely by following it with motorized stages. This method permits not only high-resolution motion analysis but also optical manipulation of neural activity [2]–[3]. In a multi-worm tracker, in contrast, multiple worms are simultaneously monitored on a single plate or multiple wells and are tracked by image-analysis software with a high throughput [4]–[7].

The imaging in these two approaches, however, has been limited to two dimensions (2-D). The approaches are therefore not adequate for studying movement behavior along all three axes, such as nictation or head lifting [8].

Here, we introduce a 3-D worm tracker (3DWT) for freely moving *C. elegans* in 3-D environments based on a stereoscopic configuration. Using the 3DWT, we provide a quantitative analysis of *C. elegans* locomotion in 3-D environments in terms of its trajectory and kinematics.

## Methods

### Principles of the 3-D worm tracker (3DWT)

**Figure 1:**
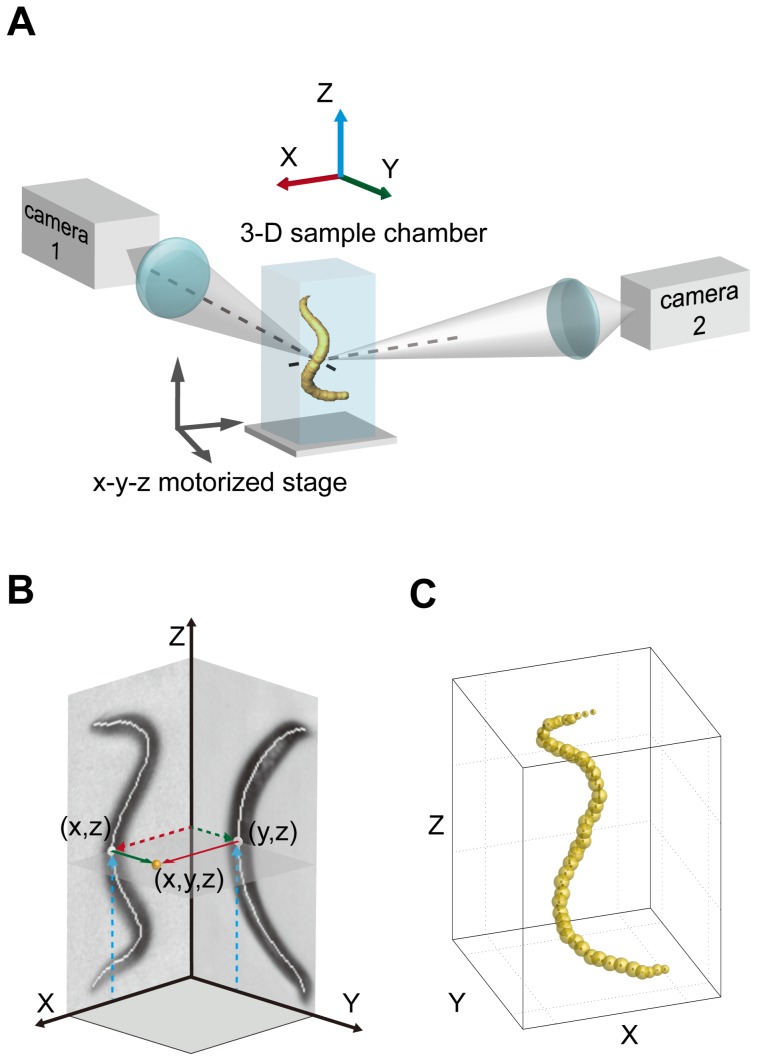
The principle of the 3-D worm tracker (3DWT). (A) Experimental scheme of the stereoscopic recording. Two imaging assemblies at right angles with the same focal point are used to synchronously record images of *C. elegans* from two perpendicular directions. (B) Stereomatching of skeletons. The two X-Z and Y-Z skeletons, extracted from the two projection images of the worm, are combined using stereomatching to reconstruct the 3-D skeleton. (C) Volume rendering of the 3-D skeleton. The 3-D volume of the worm is rendered by assigning particles that are centered on the 3D skeleton points.

The 3DWT basically consists of i) stereoscopic recording and ii) image-processing steps. Stereoscopic recording is based on two imaging assemblies at right (90°) angles with the same focal point, which allow us to synchronously image *C. elegans* from two perpendicular directions (Fig. 1A). From these two 2-D images, 3-D information such as the position or the posture of the worm is reconstructed by stereomatching (Fig. 1B) in the image-processing step.

For the 3DWT, we have two analysis strategies: i) trajectory analysis and ii) kinematic analysis. In the trajectory analysis, the worm is imaged in a field of view (25×25 mm, larger than the sample chamber) without moving the sample stage. The trajectories from two perpendicular directions, extracted from the two projection images, are then combined using stereomatching to reconstruct the 3-D trajectory. The trajectory analysis provides two parameters: the position and the moving direction of the worm in the 3-D environment.

In the kinematic analysis, the stereoscopic recording is performed at the resolutions of 4.9 and 3.1 µm/pixel (see the ‘Stereoscopic imaging system’ sub section for details) that are much smaller than the worm thickness (70∼90 µm). During the stereoscopic recording, the worm is maintained at the focal point using a three-axis motorized sample stage (Kohzu precision) that is manually controlled by using a self-developed software for multi-axis motor control (Fig. 1A). Two 2-D skeletons (Fig. 1B), extracted from two projection images of the worm, are then combined using stereomatching to reconstruct a 3-D skeleton. Finally, the 3-D volume of the worm is rendered for visualization (Fig. 1C). The kinematic analysis enables us to analyze the body-posture dynamics in a 3-D environment.

### Stereoscopic imaging system

As illustrated in Figure 1A, the stereoscopic imaging system is based on two cameras, a FASTCAM SA1.1 (Photron) with 1024×1024 pixel resolution and a PCO.1600 (PCO) with 1600×1200 pixel resolution, coupled with two identical objective lenses (×3 telecentric objective lens, Mitutoyo, NA 0.09, parfocal length 110 mm) aligned at right angles with the same focal point. The magnification of each camera could be changed by adjusting the distance between the lens and the camera. To enable synchronicity, we used a function generator (8116A, HP) that generates electrical pulses with variable frequency. Each pulse triggered image acquisitions by the two cameras, thereby enabling simultaneous imaging of a single *C. elegans* from two perpendicular directions. The imaging experiments for 3DWT were carried out in transmission mode. As a light source, we used a tungsten-halogen lamp (KWANGWOO, 150 W) with two fiber-optic bundles that guide light from the sample toward the two cameras. For the trajectory analysis, the worm was imaged at a rate of 0.5 frames/s. The field of view was adjusted to 25×25 mm, larger than the 3-D cuvette sample chamber, by using only 550×550 pixels in both cameras.

For the kinematic analysis, a stereoscopic recording was performed at resolutions of 4.9 and 3.1 µm/pixel for the FASTCAM SA1.1 and the PCO.1600, respectively, using the maximum pixel resolutions of the two cameras. The field of view (5.0×5.0 mm and 5.0×3.8 mm for the FASTCAM SA1.1 and the PCO.1600, respectively) was smaller than the 3-D sample chamber. In each imaging assembly, we also used an aperture set to f/8.8 to increase the depth of the field to ∼800 µm, which is long enough to record a worm without defocusing, as shown in Movie S1. To capture the undulatory locomotion of the worm without motion artifacts, recording was carried out with an exposure time of 16 ms and a frame rate of 13 frames/s. The images were first stored to the RAM of the cameras during the experiments and were later transferred to a computer.

### Image processing

Image analyses were performed using MATLAB (see Dataset S1). After background subtraction, two x-z and y-z images from the two directions were segmented by thresholding to obtain two binary images of the worm. To delineate the worm and the background, we set the thresholding intensity as 70% of the background intensity, the intensity of a pixel that had been randomly taken outside the worm object in the first frame of every set of sequential images. Individual pixels in an image were assigned as the worm (1) if the pixel values were smaller than the thresholding intensity and as the background (0) otherwise.

For the trajectory analysis, two centers of mass for the worm were separately calculated from the two binary images. Because each center has X-Z or Y-Z coordinates, we were able to reconstruct the X-Y-Z coordinates of the worm position by combining the coordinates of the two centers.

For the kinematic analysis, we first applied morphological closing to two binary images to clean up the spots inside the worm body [9]. The two images were then skeletonized based on a two-subiteration thinning algorithm (algorithm 1 in reference [10]). Figure 1B shows two skeletonized images overlapped on their raw images. To reconstruct a 3-D skeleton, we merged two skeletal points, (x, z) and (y, z), with a same Z-coordinate into one skeletal point (x, y, z) in 3-D (see Materials and Methods for detailed information). Similar to the particle model of the nematode [11], the 3-D volume of the worm was rendered by assigning particles that are centered on the 3-D skeleton points (Fig. 1C and see Materials and Methods). The volume-rendered images were visualized using Amira software.

## Results

### 3-D trajectory analysis

The trajectory of *C. elegans* has been widely studied to understand behaviors such as chemotaxis, thermotaxis, and food-searching behaviors [2], [12]. The trajectory in 2-D systems has been understood as a combination of forward runs, reversals and turns [8], [13] that are confined to a 2-D substrate, but the worm's trajectory in a 3-D environment has yet to be studied.

**Figure 2:**
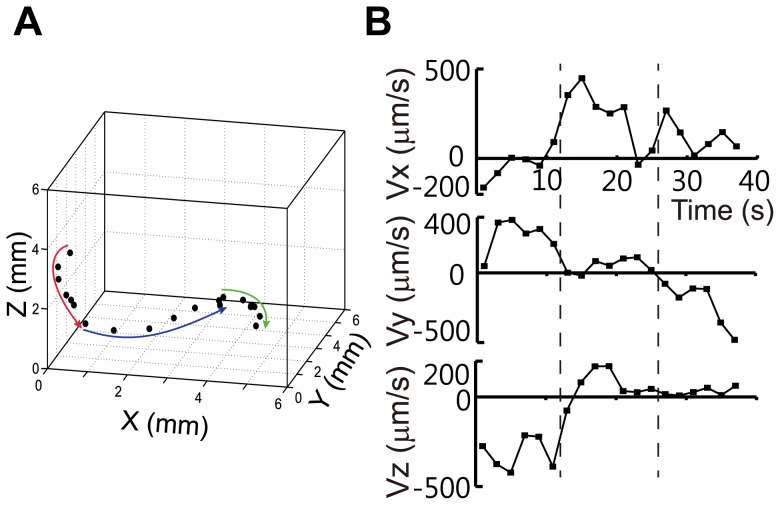
3-D trajectory and velocity of a worm. (A) 3-D trajectory of the worm. Red, blue, and green arrows are movement directions of a worm for 12 s, 14 s, and 12 s, respectively. (B) The x-, y-, and z-components of the worm's velocity.

For trajectory analysis, we tested 20 worms (average recording length: 3288 s). Using our trajectory analysis strategy, we observed that all of the worms tested moved freely in 3-D, as representatively depicted in Figure 2A. We analyzed the position of a worm moving in a 3-D sample chamber. The black solid circles represent the positions of the worm in the 3-D geometry over a total of 38 s, with images taken at 2 s intervals. The worm initially moved almost along the Y-Z plane for the first 12 s (red arrow). The worm then significantly changed its direction of movement to the positive X-direction for the next 14 s (blue arrow) and finally to the negative Y-direction for the last 12 s (green arrow) (Movie S2). This result shows that the worm shown in Figure 2 has a 3-D trajectory, not one that is restricted to a plane.

To quantitatively measure the 3-D trajectory, the velocity of the worm was estimated from its displacement every 2 s, as demonstrated in Figure 2B. The large Y- and Z-components of the velocity and the small X-components for the first 12 s indicate that the worm moved almost along the Y-Z plane, specifically with an average velocity that was only 4.4° from the Y-Z plane. For the next 14 s, the direction of movement shifted to the X-direction, with an average velocity that deviated by 82° from the previous average velocity. For the last 12 s, the direction again shifted, this time to the negative Y-direction. We note that the average velocity for the last period deviated by 50° from the plane defined by the two previous average velocities. This quantitative analysis of the movement of a representative worm shows the utility of 3DWT for studying worm behaviors in 3-D.

### 3-D kinematic analysis based on 3-D visualization

**Figure 3:**
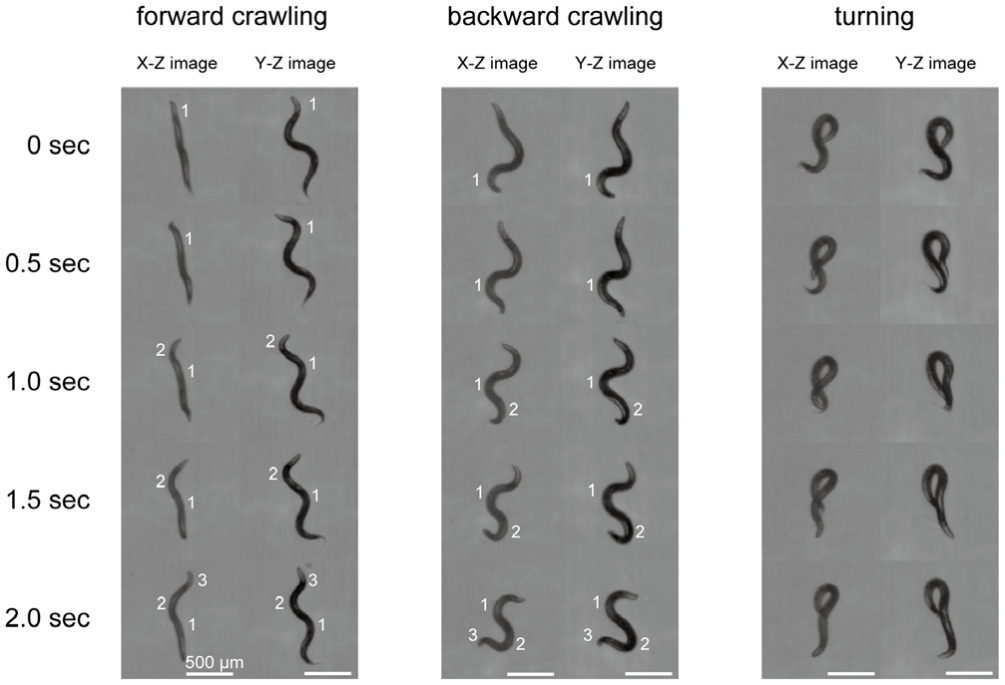
Motion of a worm in a 3-D environment. Stereoscopic images of a worm crawling forward, crawling backward, and turning. The numbers indicate bends in order. Scale bars represent 500 µm.

Our kinematic analysis using the 3DWT enables us to obtain stereoscopic images of *C. elegans* moving in 3-D environments. Figure 3 shows representative images of a worm during forward crawling, backward crawling, and turning in two different directions. Notably, bends, marked by numbers, can be separately resolved in the two directions. The stereoscopic images clearly show that the worm can have a 3-D posture that consists of bends in various directions in 3-D, as shown, for instance, by the different bending directions of bends 1 and 2 at 1.0 s in the forward crawling X-Z and Y-Z images. We also observed that the worm moves forward or backward by propagating the bends from the anterior to the posterior or vice versa, as shown by the number sequence (Movie S3). This result shows that the three locomotory modes of the worm motion in 2-D agar plates are also observed in 3-D environments.

**Figure 4:**
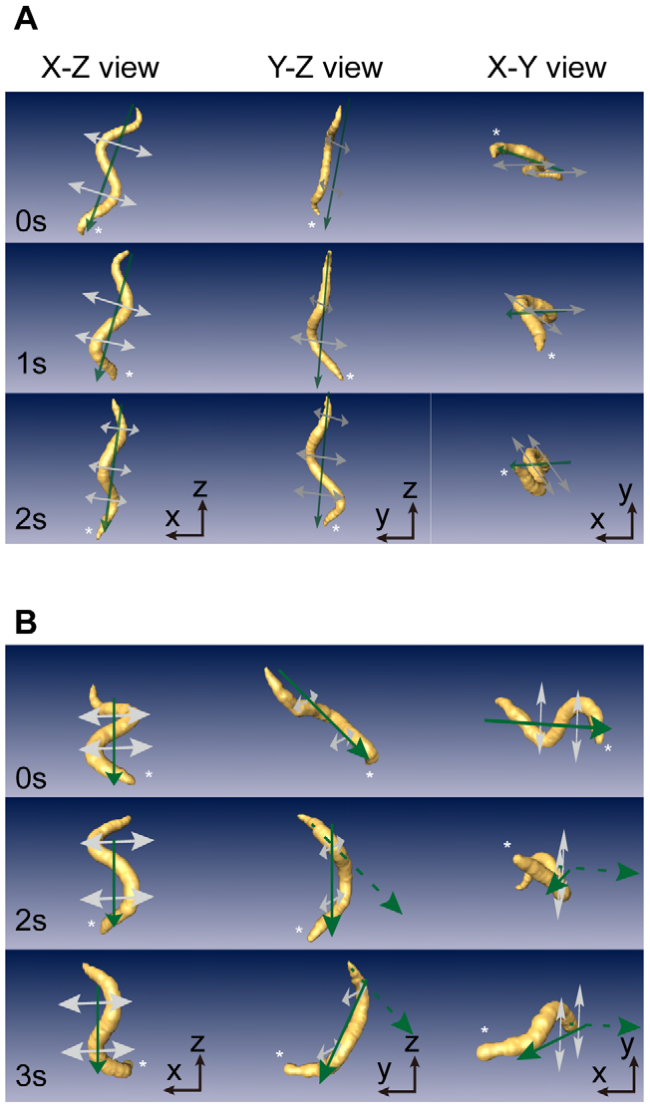
Volume-rendered images in three views. (A) 3-D posture of a worm showing bends in various directions (gray arrows) with only slight changes in the moving direction (green arrow) during crawling. (B) 3-D posture of another worm showing a significant change in the moving direction (green arrow). The asterisk in each image indicates the head of the worm.

To confirm the utility of the 3DWT in studying worm movements in 3-D, we show two different cases of worm movements in volume-rendered images in Figure 4A and B. For instance, the worm shown in Figure 4A exhibited bends in various directions (gray arrows), while the moving direction (green arrow) barely changed. At 0 s, the two bends of the worm were positioned on a plane that was almost perpendicular to the Y-Z plane. At 1 s, bending at the anterior end of the worm significantly deviated from the plane while that of the posterior bend did not (Fig. 4A: see the Y-Z view). This shows that the two bends were in different planes, indicating that the worm had a 3-D posture. At 2 s, all of the bends lay almost on another plane that was significantly inclined from that at 0 s (Fig. 4A: see the Y-Z and X-Y views and see also Movie S4). As another specific case, the worm in Figure 4B showed bends that were almost parallel as its moving direction significantly changed. At 0 s, the S-shaped worm lay on a plane. The moving direction deviated from this plane by approximately 40° at 2 s and by 80° at 3 s (see Movie S5). These two cases show the utility of the 3DWT for extracting and visualizing the posture of worms freely moving in 3-D, even though the bending and movement direction change with high complexity.

The 3DWT for kinematic analysis has several limitations. First, if a body part is occluded by another part in the projection images, the overlapping parts cannot be correctly analyzed by using two camera images (Fig. S2A). In our study, 40±15% (mean ± SD) of frames showed such overlaps in the 20 movies (average recording length: 61 s) that were investigated in kinematic analysis. The overlapped frames were manually excluded from the raw images before computational process. Despite the exclusion, an overall understanding of the worm's kinematics was obtainable by using the other frames (60±15%; mean ± SD) with no occlusion (see Movie S6). Second, if a worm's bend lies in the plane defined by the directions of the two cameras, the bend cannot be reconstructed in the 3DWT kinematic analysis. However, unlike the worm's movements in 2-D restricted substrates, cases of a worm with a bend lying in the plane were very rare (4±5%; mean ± SD) in the 3-D environment.

### 3-D kinematic analysis based on a bending vector

**Figure 5:**
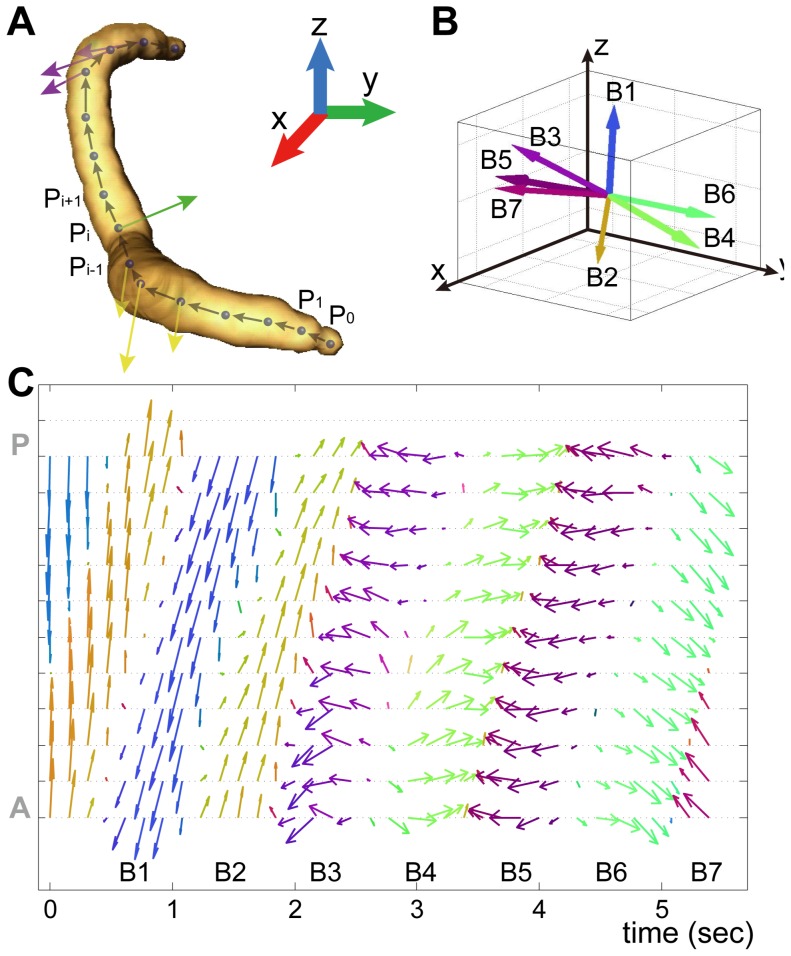
Bending vector analysis of worm motion. (A) Schematic of the bending vector in a 3-D skeleton. The arrows indicate the bending vector at a skeletal point, P_i_. (B) Variable bending vectors of a second skeletal point from the head of a representative worm during crawling. (C) The X-Y projections of bending vectors along the whole body for 5.5 s of crawling. The colors of arrows represent the directions of the bending vectors by linearly converting the components of normalized bending vectors (−1.0∼1.0) to 8-bit colors (0∼255): the X-component is displayed in red (R), the Y-component in green (G), and the Z-component in blue (B).

To quantitatively analyze the movement of *C. elegans* in 3-D, we suggest a new parameter: the bending vector. Figure 5A shows a 3-D volume-rendered image of a worm based on assigning particles that were centered on the 3-D skeleton points (Fig. 1C). The vector from P_i_ to P_i+1_ was designated as a skeleton point vector. Here, we defined the “bending vector” at a skeleton point as a vector with a length equal to the normalized magnitude of curvature (the computation is further described in “Materials and Methods”) and the direction perpendicular to the plane of bending that contains two neighboring skeleton point vectors. We presented the direction of the bending vector by the color assigned by the three vector components of the normalized bending vector: the X-component is displayed in red (R), the Y-component in green (G), and the Z-component in blue (B). Here, the vector components from −1.0 to 1.0 were linearly converted to 8-bit colors from 0 to 255. For instance, the bending vectors at the anterior, middle, and posterior parts of the body in Figure 5A are displayed by yellow, green, and purple arrows, corresponding to a negative Z-component (lack of blue), a large Y-component (green), and a negative Y-component (lack of green), respectively (see the ‘Bending vector analysis’ in the “Materials and Methods” section for details).

Figure 5B illustrates the bending vector of the second skeletal point from the head of a worm during crawling. We note variable bending vectors (B1 → B2 → B3, etc.) in 3-D with time, indicating the complexity of the worm's movement. In contrast, the vector directions in 2-D approaches are always perpendicular to the 2-D substrate as long as the plane of the bends is confined to the substrate. Figure 5C represents the X-Y projections of the bending vectors along the whole body of the worm shown in Figure 5B while it crawled for 5.5 s. We observed that the bending vector at the anterior part of the body propagated to the posterior part while maintaining the curvature (arrow length) and the direction (arrow color), representing the propagation of an undulatory wave form from the anterior to posterior end of the worm. We also analyzed bending vectors in other cases from Movie S1 and observed similar propagation patterns of undulatory waves (Figs. S2B, C, D). These results show that the bending vector defined in this study, which contains information on the bending direction and the body curvature, is a simple and useful analytic parameter for understanding the kinematics of worms in 3-D environments.

## Discussion

Despite significant technological improvements in studying *C. elegans* behavior, previous studies have been limited to behavior in 2-D substrates. We developed a 3-D worm tracker based on a stereoscopic configuration and quantitatively analyzed the locomotion of *C. elegans* in a 3-D environment. We showed that our 3DWT was able to monitor a worm's 3-D trajectory with three degrees of freedom and 3-D posture, although rigorous experiments are required to draw conclusions about worm behaviors in 3-D environments.

The strength of the 3DWT is its applicability to behavioral studies of *C. elegans* under conditions similar to its natural environment, based on quantitative analyses of the trajectory and body posture. The 3DWT can also be applied to biomechanical studies in which the estimation of external forces is crucial to mechanically analyzing the animals' kinematics.

Our current 3DWT is not yet optimal for real-time analysis because of many image-processing steps. In the future, we expect to be able to track a worm in real-time by optimizing the imaging systems and the computational processes. Despite these limitations, to our knowledge, our 3DWT is the first quantitative analytic method for monitoring worm behaviors in a 3-D environment. The 3DWT affords new opportunities for understanding the nervous system function that regulates an animal's behavior in natural 3-D environments.

## Materials and Methods

### Sample preparation

Gelatin (SIGMA G-8150) was dissolved in M9 buffer at a 2.5% concentration and placed in a cuvette (1 cm×1 cm×1.5 cm). Young adult (day 1) wild type N2 *C. elegans* worms were grown under standard conditions, i.e., on an agar plate at 20°C in the presence of food, *E. coli* strain OP50 [14]. For stereoscopic imaging experiments, young adult hermaphrodites were placed in the cuvette using a platinum wire worm pick. Initially the worms stayed in the grooves made in the gelatin using a worm pick for 16±6 s (n = 20). Imaging experiments started after the worms had burrowed into the gelatin from the groove.

### Stereomatching algorithm for kinematic analysis

First, we extracted from head to tail the X-Z and Y-Z coordinates of the skeletal points from two X-Z and Y-Z skeletons (Fig. S1A) of a worm, as shown by the X-Z and Y-Z matrices in Figure S1C. The Z-coordinates of the first rows in both X-Z and Y-Z matrices were not always the same, mostly due to errors in image processing. The mismatched top rows in either matrix were excluded from 3-D reconstruction. Specifically, the mismatched rows were searched by evaluating two conditions as follows:

**A.**

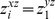



**B.**

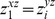



where 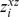 and 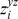 are the Z-coordinates of the 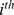 rows in the X-Z and Y-Z matrices, respectively. If the **condition A** was first satisfied while increasing the index 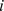 from 1, the top (*i-1*) rows in the X-Z matrix were excluded. Otherwise, the top (*i-1*) rows in the Y-Z matrix were excluded.

Next, we carried out stereomatching based on merging one X-Z and one Y-Z rows with a same Z-coordinate into one X-Y-Z coordinate. If two or more consecutive rows had a same Z-coordinate in the X-Z or Y-Z matrix (for instance, the light gray rows in the blue box of the X-Z matrix in Fig. S1C), the consecutive rows were represented by one row with their average X-Z or Y-Z coordinates. In addition, if there were mismatched rows of which the Z-coordinates were absent in the other matrix (for instance, the green rows in Fig. S1C), again due to errors in image processing, their Z-coordinates were represented by that of the nearest matched row (for instance, the blue rows in the Y-Z matrix (Fig. S1C)).

The computation of stereomatching started from a pair of the first matched X-Z and Y-Z rows with a same Z-coordinate in the matrices. Then, the X-Z and Y-Z sets of consecutive rows with the Z-coordinate of the pair were searched. The two sets including the pair were merged into one X-Y-Z row with their average X, Y, and Z coordinates, if the Z-coordinates of the X-Z and Y-Z rows next to the sets were the same. If not, i.e. if mismatched in Z-coordinates, we first searched the nearest next matched rows by evaluating two conditions as below:

**A.**

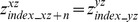



**B.**

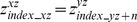



where 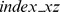 and 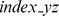 are the row-indices of the first mismatched X-Z and Y-Z rows. If the **condition A** was first satisfied while increasing n from 1, the 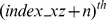 X-Z and the 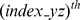 Y-Z rows were the nearest next matched rows. Otherwise, the 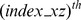 X-Z and the 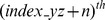 Y-Z. Then, the two matched sets and the mismatched rows including the starting pair were merged into one X-Y-Z row with their average X-Y coordinates and the Z-coordinate of the starting pair. We repeated the stereomatching process by taking the next matched pair as a new starting pair until the last matched rows of the two matrices.

### Particle radius estimation

To estimate the particle radius at each skeleton point, we performed an additional 2-D imaging experiment for a worm crawling on a standard NGM plate without food. 2-D images were taken by using the same PCO.1600 imaging setup for kinematic analysis while vertically mounting the plate. By taking one in the 2-D images obtained, we carried out the same segmentation and skeletonization processes. The particle radius at each skeletal point was then estimated by measuring the distance from the point to the nearest boundary of the segmented image.

### Bending vector analysis

To analyze the worm kinematics, the 3-D skeleton of the worm was divided into 13 sections. The magnitude of the bending vector, the curvature, was defined as the directional difference between neighboring skeleton point vectors divided by the distance between the neighboring skeleton points. The direction of the bending vector was defined as the direction of the cross product of two neighboring skeleton point vectors, i.e., the direction normal to the plane including the two skeleton point vectors. The length of the arrow that represents the bending vector was estimated by normalizing a high curvature value, 45°/100 µm, to 1 in our experiments, multiplying curvature values by 100/45. The color of the arrow, which represents the direction of the bending vector, was calculated by linearly converting the components of normalized bending vectors (−1.0∼1.0) to 8-bit colors (0∼255) as given below.
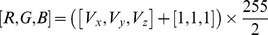



Here, R, G, and B are the 8-bit colors and Vx, Vy, and Vz are the x, y, and z components of the normalized bending vector. The bending vectors were plotted using MATLAB.

## Supporting Information

Figure S1 **Stereomatching process for kinematic analysis.** (A) Two skeletons of a worm overlapped on raw images. (B) Two views of volume rendered image reconstructed from the worm in (A) a 3-D reconstructed worm. The resulting images show that our reconstruction of the worm was properly performed. (C) Stereomatching of two skeletons of a worm. The left two X-Z and Y-Z matrices were extracted from head to tail the X-Z and Y-Z coordinates of the skeletal points from (A) and the X-Y-Z matrix on the right was merged from the two matrices. The yellow, blue and pink sections correspond to the skeletal points in the yellow, blue and pink boxes of the worm shown in (A), respectively.
(TIF)

Figure S2 **Representative results analyzed from a movie using 3DWT.** (A) Two stereoscopic images of a worm showing overlapping body parts (White box shows overlapping region). (B), (C) and (D) show bending vectors obtained from a movie (Movie S1) without occlusion.
(TIF)

Movie S1 **Stereoscopic movie of a worm moving freely in 3-D.**
(MP4)

Movie S2 **Trajectory of a worm.**
(MP4)

Movie S3 **Stereoscopic movie of a worm showing forward crawling, backward crawling and turning.**
(MP4)

Movie S4 **3-D movie of a worm in** **Figure 4A** **.**
(MP4)

Movie S5 **3-D movie of a worm in** **Figure 4B** **.**
(MP4)

Movie S6 **3-D movie of a worm tracked for a minute.**
(MP4)

Dataset S1 **MATLAB-based code for kinematic analysis of 3-D worm tracker.** The *.zip file contains *.m files for reconstruction and visualization of a stereoscopic image set. It also contains sample images (30 frames with 1 sec intervals), and an CSV file including width at each region of a worm. The MATLAB based code requires MATLAB and Image Processing Toolbox.
(ZIP)
