# Supplementary figures and images for: 3-D Worm Tracker for Freely Moving C. elegans
Source: PLoS One. 2013 Feb 21;8(2):e57484. doi: 10.1371/journal.pone.0057484 (PMC3578814; doi:10.1371/journal.pone.0057484)

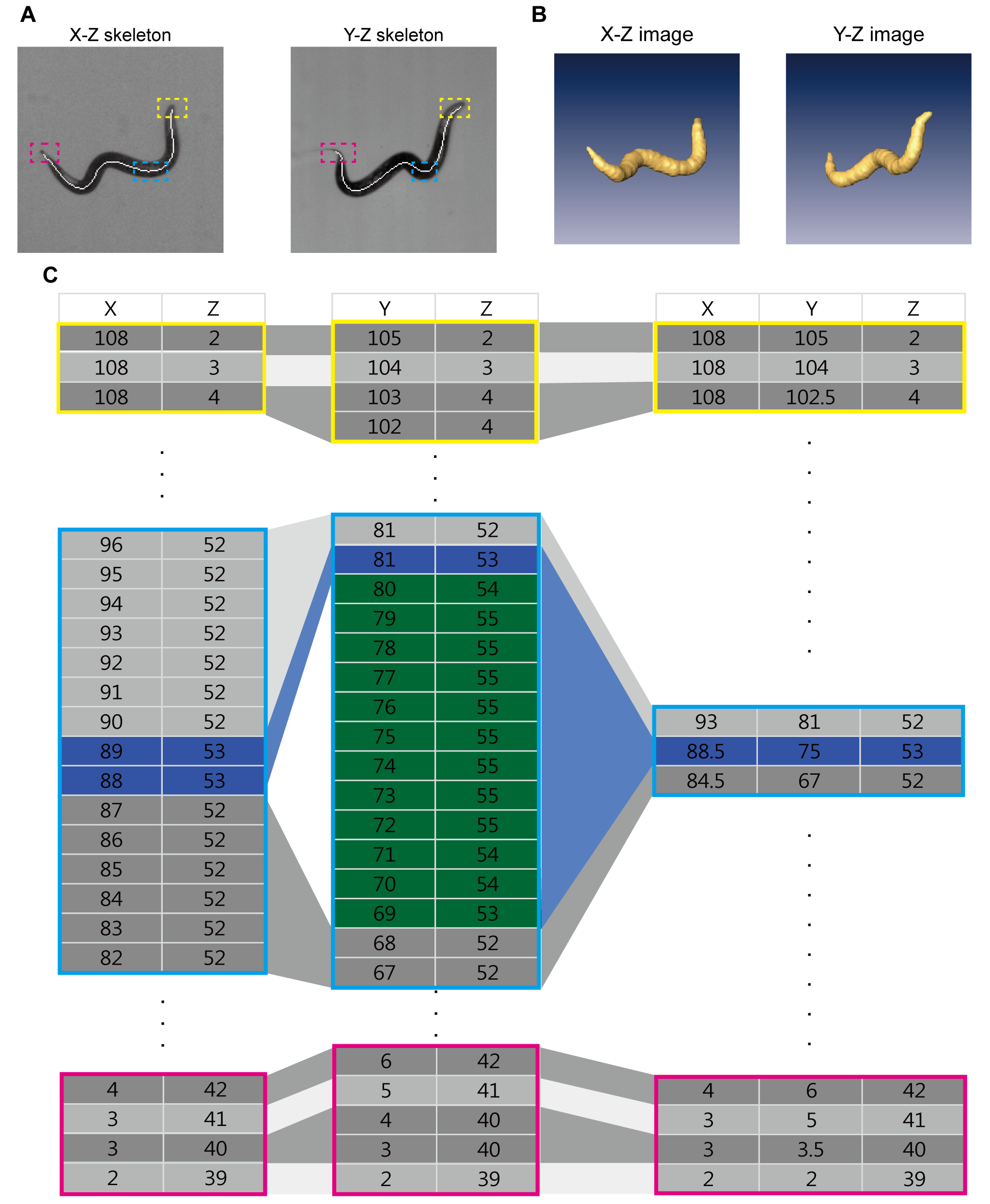

Supplement: Figure S1 — Stereomatching process for kinematic analysis. (A) Two skeletons of a worm overlapped on raw images. (B) Two views of volume rendered image reconstructed from the worm in (A) a 3-D reconstructed worm. The resulting images show that our reconstruction of the worm was properly performed. (C) Stereomatching of two skeletons of a worm. The left two X-Z and Y-Z matrices were extracted from head to tail the X-Z and Y-Z coordinates of the skeletal points from (A) and the X-Y-Z matrix on the right was merged from the two matrices. The yellow, blue and pink sections correspond to the skeletal points in the yellow, blue and pink boxes of the worm shown in (A), respectively. (TIF) [file pone.0057484.s001.tif]

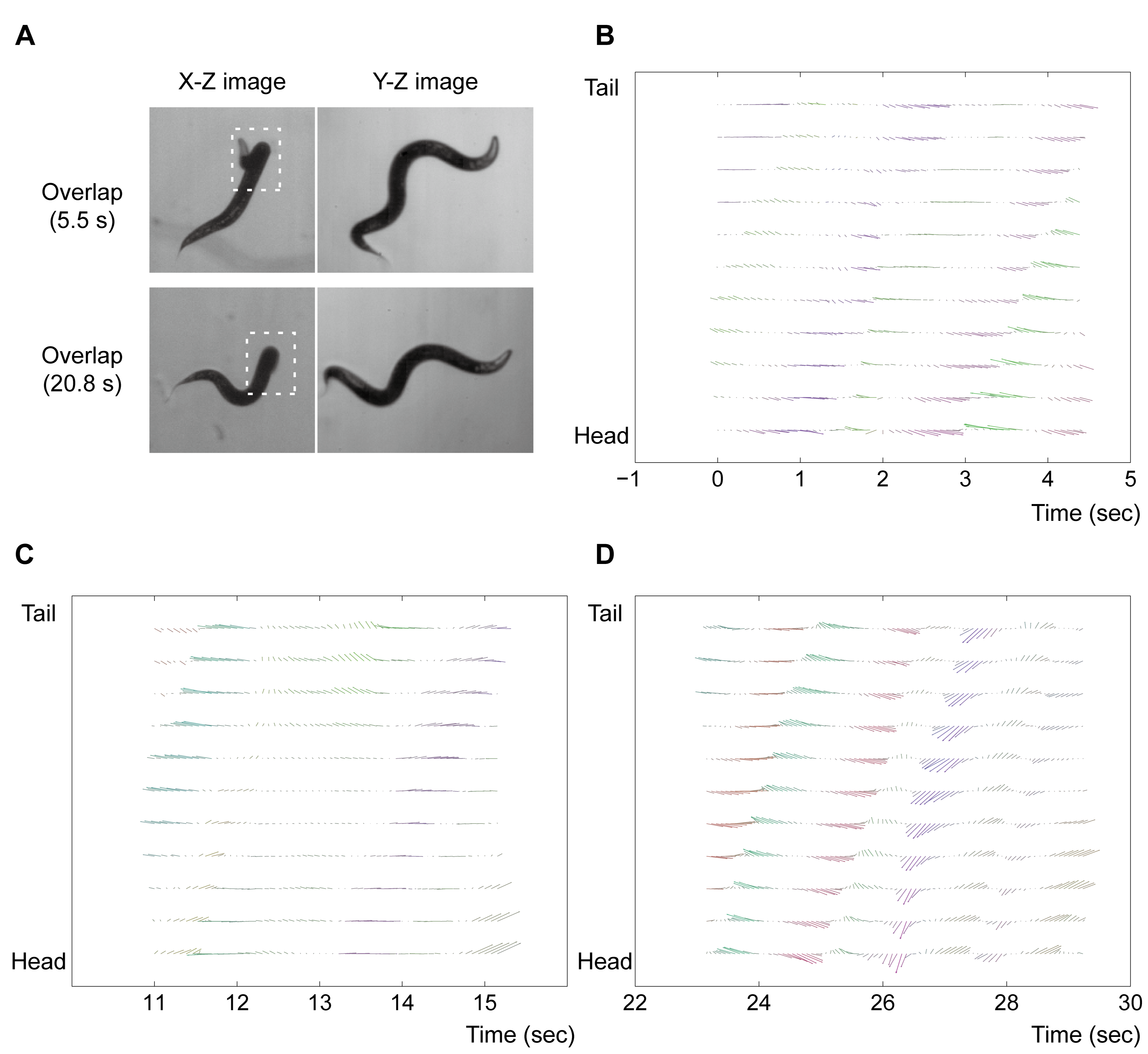

Supplement: Figure S2 — Representative results analyzed from a movie using 3DWT. (A) Two stereoscopic images of a worm showing overlapping body parts (White box shows overlapping region). (B), (C) and (D) show bending vectors obtained from a movie (Movie S1) without occlusion. (TIF) [file pone.0057484.s002.tif]

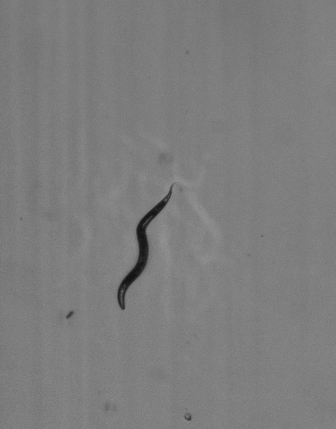

Supplement: Dataset S1 — MATLAB-based code for kinematic analysis of 3-D worm tracker. The *.zip file contains *.m files for reconstruction and visualization of a stereoscopic image set. It also contains sample images (30 frames with 1 sec intervals), and an CSV file including width at each region of a worm. The MATLAB based code requires MATLAB and Image Processing Toolbox. (ZIP) [file pone.0057484.s009.zip › 3D worm tracker code/sample/1_1.tif]

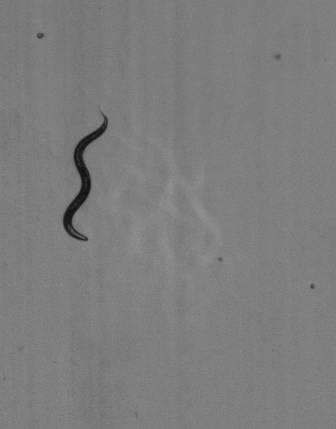

Supplement: Dataset S1 — MATLAB-based code for kinematic analysis of 3-D worm tracker. The *.zip file contains *.m files for reconstruction and visualization of a stereoscopic image set. It also contains sample images (30 frames with 1 sec intervals), and an CSV file including width at each region of a worm. The MATLAB based code requires MATLAB and Image Processing Toolbox. (ZIP) [file pone.0057484.s009.zip › 3D worm tracker code/sample/1_10.tif]

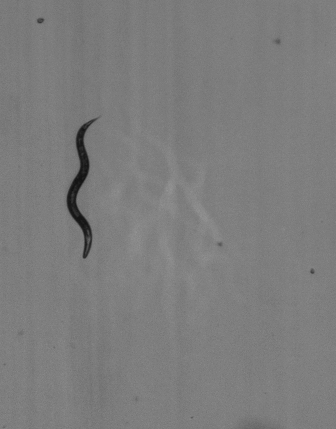

Supplement: Dataset S1 — MATLAB-based code for kinematic analysis of 3-D worm tracker. The *.zip file contains *.m files for reconstruction and visualization of a stereoscopic image set. It also contains sample images (30 frames with 1 sec intervals), and an CSV file including width at each region of a worm. The MATLAB based code requires MATLAB and Image Processing Toolbox. (ZIP) [file pone.0057484.s009.zip › 3D worm tracker code/sample/1_11.tif]

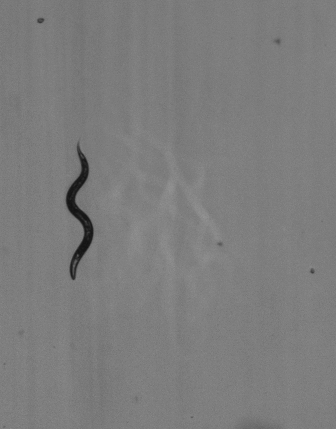

Supplement: Dataset S1 — MATLAB-based code for kinematic analysis of 3-D worm tracker. The *.zip file contains *.m files for reconstruction and visualization of a stereoscopic image set. It also contains sample images (30 frames with 1 sec intervals), and an CSV file including width at each region of a worm. The MATLAB based code requires MATLAB and Image Processing Toolbox. (ZIP) [file pone.0057484.s009.zip › 3D worm tracker code/sample/1_12.tif]

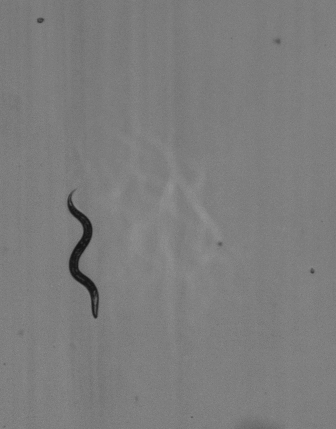

Supplement: Dataset S1 — MATLAB-based code for kinematic analysis of 3-D worm tracker. The *.zip file contains *.m files for reconstruction and visualization of a stereoscopic image set. It also contains sample images (30 frames with 1 sec intervals), and an CSV file including width at each region of a worm. The MATLAB based code requires MATLAB and Image Processing Toolbox. (ZIP) [file pone.0057484.s009.zip › 3D worm tracker code/sample/1_13.tif]

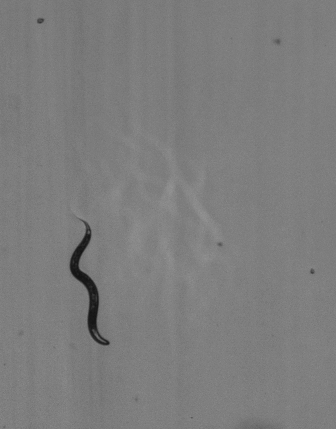

Supplement: Dataset S1 — MATLAB-based code for kinematic analysis of 3-D worm tracker. The *.zip file contains *.m files for reconstruction and visualization of a stereoscopic image set. It also contains sample images (30 frames with 1 sec intervals), and an CSV file including width at each region of a worm. The MATLAB based code requires MATLAB and Image Processing Toolbox. (ZIP) [file pone.0057484.s009.zip › 3D worm tracker code/sample/1_14.tif]

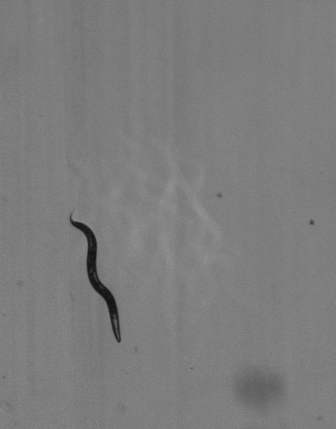

Supplement: Dataset S1 — MATLAB-based code for kinematic analysis of 3-D worm tracker. The *.zip file contains *.m files for reconstruction and visualization of a stereoscopic image set. It also contains sample images (30 frames with 1 sec intervals), and an CSV file including width at each region of a worm. The MATLAB based code requires MATLAB and Image Processing Toolbox. (ZIP) [file pone.0057484.s009.zip › 3D worm tracker code/sample/1_15.tif]

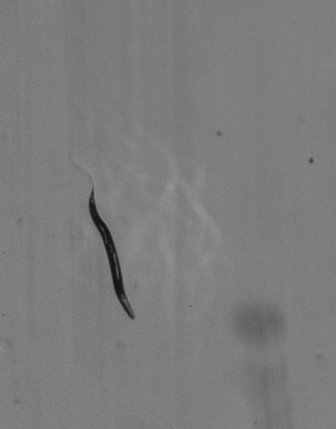

Supplement: Dataset S1 — MATLAB-based code for kinematic analysis of 3-D worm tracker. The *.zip file contains *.m files for reconstruction and visualization of a stereoscopic image set. It also contains sample images (30 frames with 1 sec intervals), and an CSV file including width at each region of a worm. The MATLAB based code requires MATLAB and Image Processing Toolbox. (ZIP) [file pone.0057484.s009.zip › 3D worm tracker code/sample/1_16.tif]

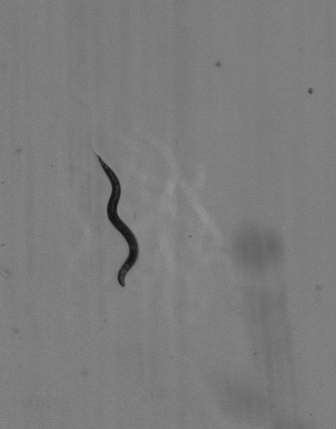

Supplement: Dataset S1 — MATLAB-based code for kinematic analysis of 3-D worm tracker. The *.zip file contains *.m files for reconstruction and visualization of a stereoscopic image set. It also contains sample images (30 frames with 1 sec intervals), and an CSV file including width at each region of a worm. The MATLAB based code requires MATLAB and Image Processing Toolbox. (ZIP) [file pone.0057484.s009.zip › 3D worm tracker code/sample/1_17.tif]

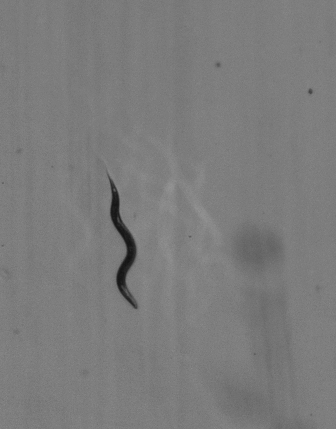

Supplement: Dataset S1 — MATLAB-based code for kinematic analysis of 3-D worm tracker. The *.zip file contains *.m files for reconstruction and visualization of a stereoscopic image set. It also contains sample images (30 frames with 1 sec intervals), and an CSV file including width at each region of a worm. The MATLAB based code requires MATLAB and Image Processing Toolbox. (ZIP) [file pone.0057484.s009.zip › 3D worm tracker code/sample/1_18.tif]

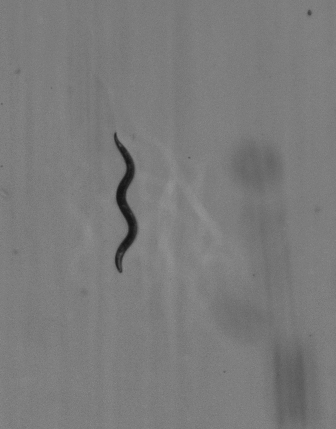

Supplement: Dataset S1 — MATLAB-based code for kinematic analysis of 3-D worm tracker. The *.zip file contains *.m files for reconstruction and visualization of a stereoscopic image set. It also contains sample images (30 frames with 1 sec intervals), and an CSV file including width at each region of a worm. The MATLAB based code requires MATLAB and Image Processing Toolbox. (ZIP) [file pone.0057484.s009.zip › 3D worm tracker code/sample/1_19.tif]

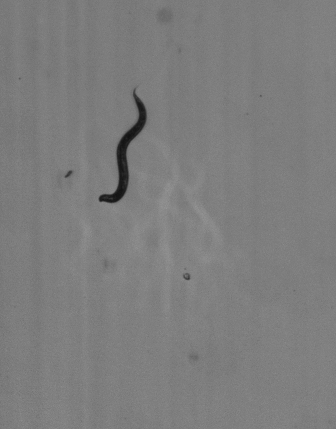

Supplement: Dataset S1 — MATLAB-based code for kinematic analysis of 3-D worm tracker. The *.zip file contains *.m files for reconstruction and visualization of a stereoscopic image set. It also contains sample images (30 frames with 1 sec intervals), and an CSV file including width at each region of a worm. The MATLAB based code requires MATLAB and Image Processing Toolbox. (ZIP) [file pone.0057484.s009.zip › 3D worm tracker code/sample/1_2.tif]

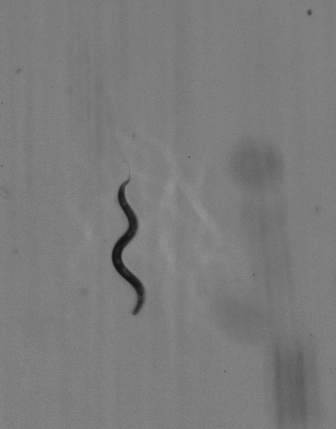

Supplement: Dataset S1 — MATLAB-based code for kinematic analysis of 3-D worm tracker. The *.zip file contains *.m files for reconstruction and visualization of a stereoscopic image set. It also contains sample images (30 frames with 1 sec intervals), and an CSV file including width at each region of a worm. The MATLAB based code requires MATLAB and Image Processing Toolbox. (ZIP) [file pone.0057484.s009.zip › 3D worm tracker code/sample/1_20.tif]

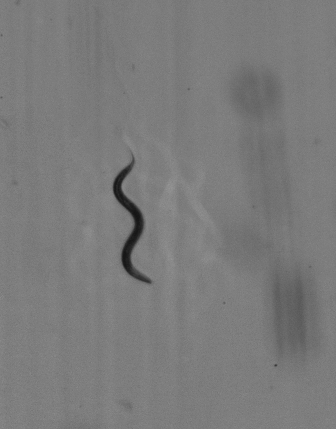

Supplement: Dataset S1 — MATLAB-based code for kinematic analysis of 3-D worm tracker. The *.zip file contains *.m files for reconstruction and visualization of a stereoscopic image set. It also contains sample images (30 frames with 1 sec intervals), and an CSV file including width at each region of a worm. The MATLAB based code requires MATLAB and Image Processing Toolbox. (ZIP) [file pone.0057484.s009.zip › 3D worm tracker code/sample/1_21.tif]

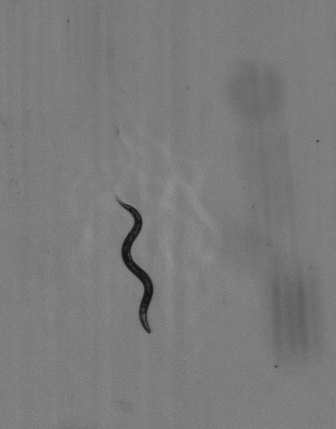

Supplement: Dataset S1 — MATLAB-based code for kinematic analysis of 3-D worm tracker. The *.zip file contains *.m files for reconstruction and visualization of a stereoscopic image set. It also contains sample images (30 frames with 1 sec intervals), and an CSV file including width at each region of a worm. The MATLAB based code requires MATLAB and Image Processing Toolbox. (ZIP) [file pone.0057484.s009.zip › 3D worm tracker code/sample/1_22.tif]

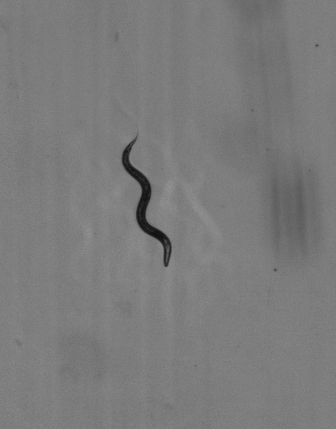

Supplement: Dataset S1 — MATLAB-based code for kinematic analysis of 3-D worm tracker. The *.zip file contains *.m files for reconstruction and visualization of a stereoscopic image set. It also contains sample images (30 frames with 1 sec intervals), and an CSV file including width at each region of a worm. The MATLAB based code requires MATLAB and Image Processing Toolbox. (ZIP) [file pone.0057484.s009.zip › 3D worm tracker code/sample/1_23.tif]

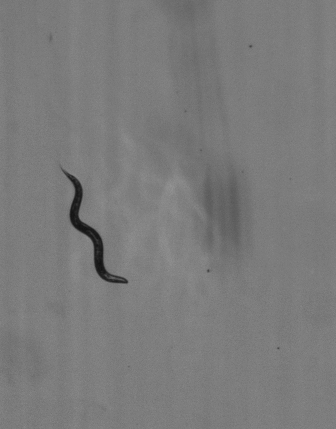

Supplement: Dataset S1 — MATLAB-based code for kinematic analysis of 3-D worm tracker. The *.zip file contains *.m files for reconstruction and visualization of a stereoscopic image set. It also contains sample images (30 frames with 1 sec intervals), and an CSV file including width at each region of a worm. The MATLAB based code requires MATLAB and Image Processing Toolbox. (ZIP) [file pone.0057484.s009.zip › 3D worm tracker code/sample/1_24.tif]

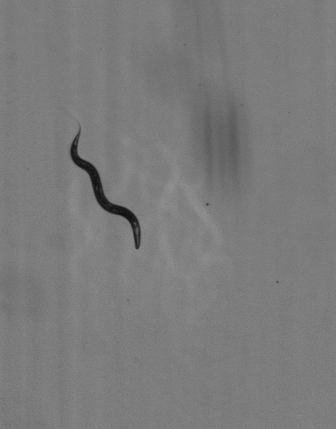

Supplement: Dataset S1 — MATLAB-based code for kinematic analysis of 3-D worm tracker. The *.zip file contains *.m files for reconstruction and visualization of a stereoscopic image set. It also contains sample images (30 frames with 1 sec intervals), and an CSV file including width at each region of a worm. The MATLAB based code requires MATLAB and Image Processing Toolbox. (ZIP) [file pone.0057484.s009.zip › 3D worm tracker code/sample/1_25.tif]

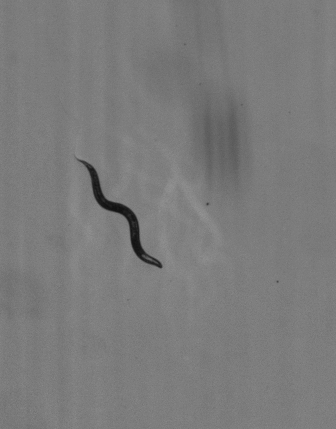

Supplement: Dataset S1 — MATLAB-based code for kinematic analysis of 3-D worm tracker. The *.zip file contains *.m files for reconstruction and visualization of a stereoscopic image set. It also contains sample images (30 frames with 1 sec intervals), and an CSV file including width at each region of a worm. The MATLAB based code requires MATLAB and Image Processing Toolbox. (ZIP) [file pone.0057484.s009.zip › 3D worm tracker code/sample/1_26.tif]

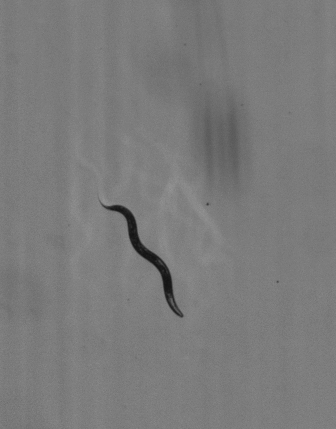

Supplement: Dataset S1 — MATLAB-based code for kinematic analysis of 3-D worm tracker. The *.zip file contains *.m files for reconstruction and visualization of a stereoscopic image set. It also contains sample images (30 frames with 1 sec intervals), and an CSV file including width at each region of a worm. The MATLAB based code requires MATLAB and Image Processing Toolbox. (ZIP) [file pone.0057484.s009.zip › 3D worm tracker code/sample/1_27.tif]

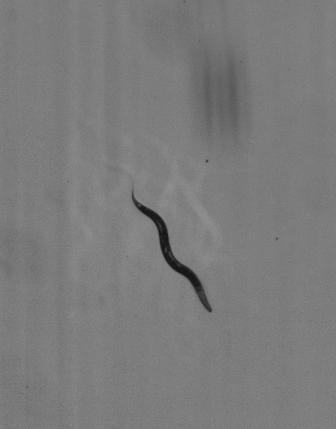

Supplement: Dataset S1 — MATLAB-based code for kinematic analysis of 3-D worm tracker. The *.zip file contains *.m files for reconstruction and visualization of a stereoscopic image set. It also contains sample images (30 frames with 1 sec intervals), and an CSV file including width at each region of a worm. The MATLAB based code requires MATLAB and Image Processing Toolbox. (ZIP) [file pone.0057484.s009.zip › 3D worm tracker code/sample/1_28.tif]

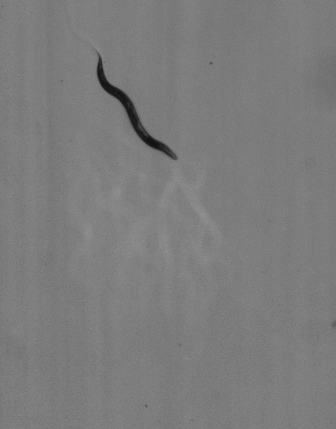

Supplement: Dataset S1 — MATLAB-based code for kinematic analysis of 3-D worm tracker. The *.zip file contains *.m files for reconstruction and visualization of a stereoscopic image set. It also contains sample images (30 frames with 1 sec intervals), and an CSV file including width at each region of a worm. The MATLAB based code requires MATLAB and Image Processing Toolbox. (ZIP) [file pone.0057484.s009.zip › 3D worm tracker code/sample/1_29.tif]

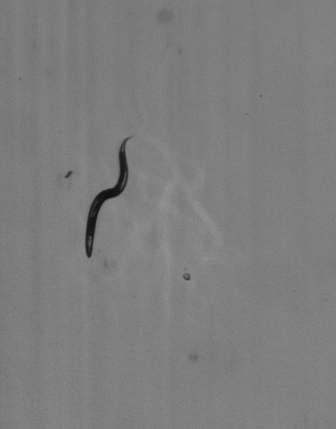

Supplement: Dataset S1 — MATLAB-based code for kinematic analysis of 3-D worm tracker. The *.zip file contains *.m files for reconstruction and visualization of a stereoscopic image set. It also contains sample images (30 frames with 1 sec intervals), and an CSV file including width at each region of a worm. The MATLAB based code requires MATLAB and Image Processing Toolbox. (ZIP) [file pone.0057484.s009.zip › 3D worm tracker code/sample/1_3.tif]

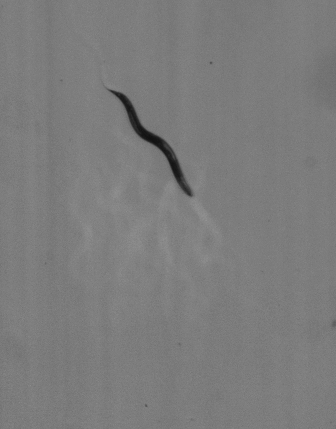

Supplement: Dataset S1 — MATLAB-based code for kinematic analysis of 3-D worm tracker. The *.zip file contains *.m files for reconstruction and visualization of a stereoscopic image set. It also contains sample images (30 frames with 1 sec intervals), and an CSV file including width at each region of a worm. The MATLAB based code requires MATLAB and Image Processing Toolbox. (ZIP) [file pone.0057484.s009.zip › 3D worm tracker code/sample/1_30.tif]

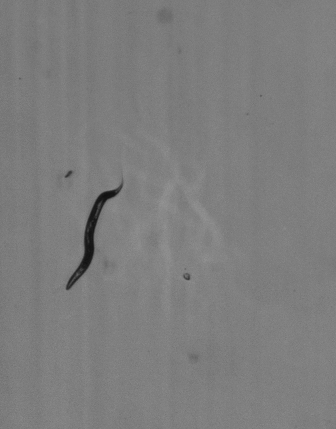

Supplement: Dataset S1 — MATLAB-based code for kinematic analysis of 3-D worm tracker. The *.zip file contains *.m files for reconstruction and visualization of a stereoscopic image set. It also contains sample images (30 frames with 1 sec intervals), and an CSV file including width at each region of a worm. The MATLAB based code requires MATLAB and Image Processing Toolbox. (ZIP) [file pone.0057484.s009.zip › 3D worm tracker code/sample/1_4.tif]

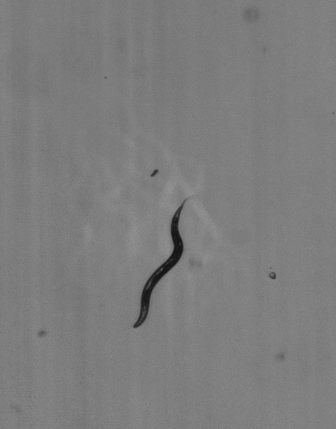

Supplement: Dataset S1 — MATLAB-based code for kinematic analysis of 3-D worm tracker. The *.zip file contains *.m files for reconstruction and visualization of a stereoscopic image set. It also contains sample images (30 frames with 1 sec intervals), and an CSV file including width at each region of a worm. The MATLAB based code requires MATLAB and Image Processing Toolbox. (ZIP) [file pone.0057484.s009.zip › 3D worm tracker code/sample/1_5.tif]

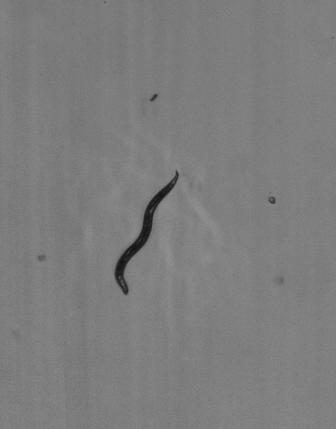

Supplement: Dataset S1 — MATLAB-based code for kinematic analysis of 3-D worm tracker. The *.zip file contains *.m files for reconstruction and visualization of a stereoscopic image set. It also contains sample images (30 frames with 1 sec intervals), and an CSV file including width at each region of a worm. The MATLAB based code requires MATLAB and Image Processing Toolbox. (ZIP) [file pone.0057484.s009.zip › 3D worm tracker code/sample/1_6.tif]

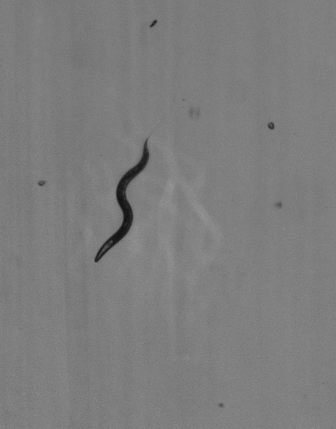

Supplement: Dataset S1 — MATLAB-based code for kinematic analysis of 3-D worm tracker. The *.zip file contains *.m files for reconstruction and visualization of a stereoscopic image set. It also contains sample images (30 frames with 1 sec intervals), and an CSV file including width at each region of a worm. The MATLAB based code requires MATLAB and Image Processing Toolbox. (ZIP) [file pone.0057484.s009.zip › 3D worm tracker code/sample/1_7.tif]

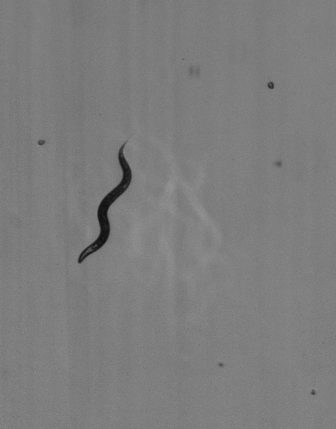

Supplement: Dataset S1 — MATLAB-based code for kinematic analysis of 3-D worm tracker. The *.zip file contains *.m files for reconstruction and visualization of a stereoscopic image set. It also contains sample images (30 frames with 1 sec intervals), and an CSV file including width at each region of a worm. The MATLAB based code requires MATLAB and Image Processing Toolbox. (ZIP) [file pone.0057484.s009.zip › 3D worm tracker code/sample/1_8.tif]

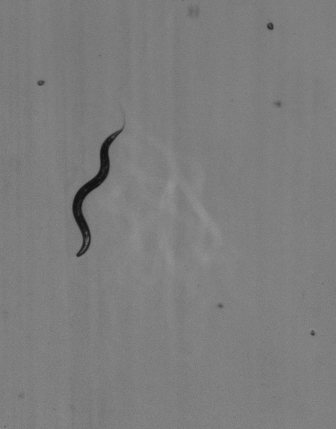

Supplement: Dataset S1 — MATLAB-based code for kinematic analysis of 3-D worm tracker. The *.zip file contains *.m files for reconstruction and visualization of a stereoscopic image set. It also contains sample images (30 frames with 1 sec intervals), and an CSV file including width at each region of a worm. The MATLAB based code requires MATLAB and Image Processing Toolbox. (ZIP) [file pone.0057484.s009.zip › 3D worm tracker code/sample/1_9.tif]

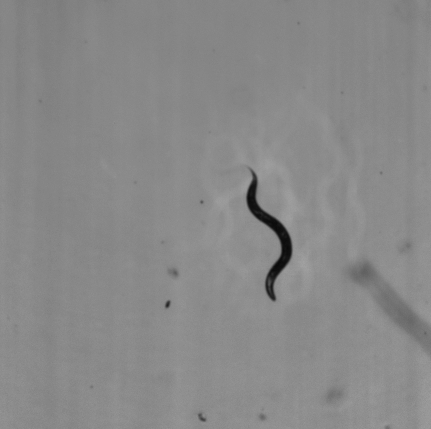

Supplement: Dataset S1 — MATLAB-based code for kinematic analysis of 3-D worm tracker. The *.zip file contains *.m files for reconstruction and visualization of a stereoscopic image set. It also contains sample images (30 frames with 1 sec intervals), and an CSV file including width at each region of a worm. The MATLAB based code requires MATLAB and Image Processing Toolbox. (ZIP) [file pone.0057484.s009.zip › 3D worm tracker code/sample/2_1.tif]

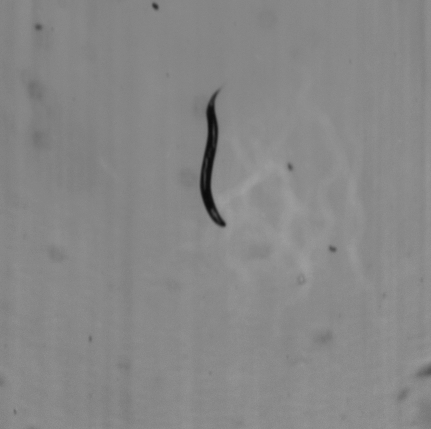

Supplement: Dataset S1 — MATLAB-based code for kinematic analysis of 3-D worm tracker. The *.zip file contains *.m files for reconstruction and visualization of a stereoscopic image set. It also contains sample images (30 frames with 1 sec intervals), and an CSV file including width at each region of a worm. The MATLAB based code requires MATLAB and Image Processing Toolbox. (ZIP) [file pone.0057484.s009.zip › 3D worm tracker code/sample/2_10.tif]

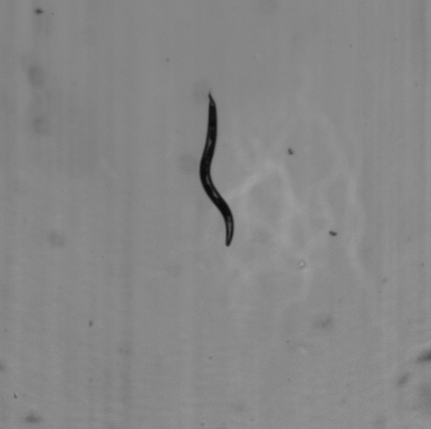

Supplement: Dataset S1 — MATLAB-based code for kinematic analysis of 3-D worm tracker. The *.zip file contains *.m files for reconstruction and visualization of a stereoscopic image set. It also contains sample images (30 frames with 1 sec intervals), and an CSV file including width at each region of a worm. The MATLAB based code requires MATLAB and Image Processing Toolbox. (ZIP) [file pone.0057484.s009.zip › 3D worm tracker code/sample/2_11.tif]

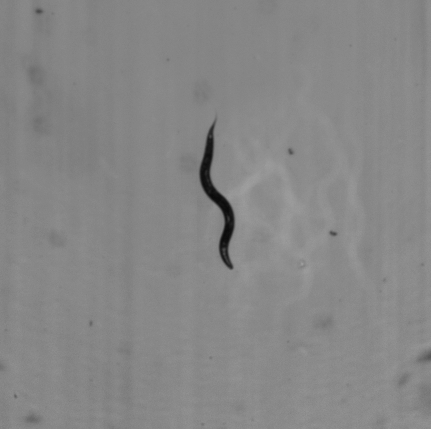

Supplement: Dataset S1 — MATLAB-based code for kinematic analysis of 3-D worm tracker. The *.zip file contains *.m files for reconstruction and visualization of a stereoscopic image set. It also contains sample images (30 frames with 1 sec intervals), and an CSV file including width at each region of a worm. The MATLAB based code requires MATLAB and Image Processing Toolbox. (ZIP) [file pone.0057484.s009.zip › 3D worm tracker code/sample/2_12.tif]

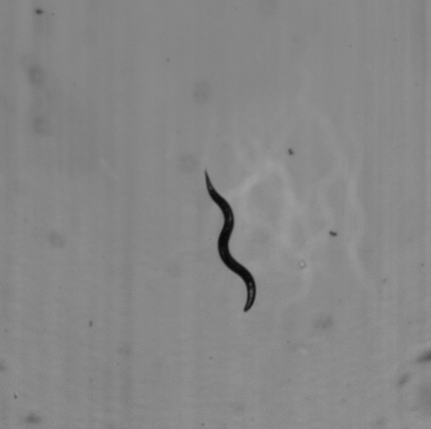

Supplement: Dataset S1 — MATLAB-based code for kinematic analysis of 3-D worm tracker. The *.zip file contains *.m files for reconstruction and visualization of a stereoscopic image set. It also contains sample images (30 frames with 1 sec intervals), and an CSV file including width at each region of a worm. The MATLAB based code requires MATLAB and Image Processing Toolbox. (ZIP) [file pone.0057484.s009.zip › 3D worm tracker code/sample/2_13.tif]

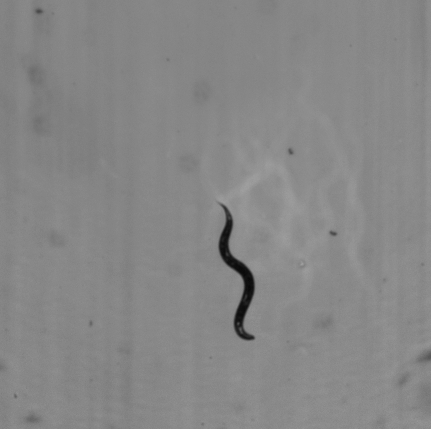

Supplement: Dataset S1 — MATLAB-based code for kinematic analysis of 3-D worm tracker. The *.zip file contains *.m files for reconstruction and visualization of a stereoscopic image set. It also contains sample images (30 frames with 1 sec intervals), and an CSV file including width at each region of a worm. The MATLAB based code requires MATLAB and Image Processing Toolbox. (ZIP) [file pone.0057484.s009.zip › 3D worm tracker code/sample/2_14.tif]

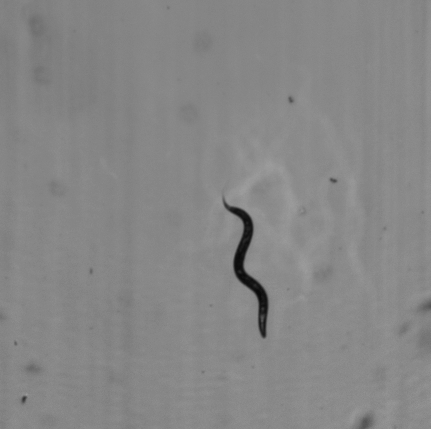

Supplement: Dataset S1 — MATLAB-based code for kinematic analysis of 3-D worm tracker. The *.zip file contains *.m files for reconstruction and visualization of a stereoscopic image set. It also contains sample images (30 frames with 1 sec intervals), and an CSV file including width at each region of a worm. The MATLAB based code requires MATLAB and Image Processing Toolbox. (ZIP) [file pone.0057484.s009.zip › 3D worm tracker code/sample/2_15.tif]

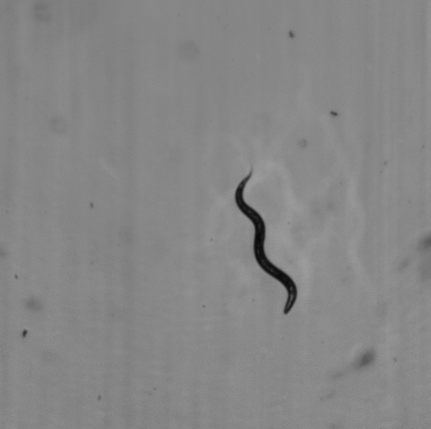

Supplement: Dataset S1 — MATLAB-based code for kinematic analysis of 3-D worm tracker. The *.zip file contains *.m files for reconstruction and visualization of a stereoscopic image set. It also contains sample images (30 frames with 1 sec intervals), and an CSV file including width at each region of a worm. The MATLAB based code requires MATLAB and Image Processing Toolbox. (ZIP) [file pone.0057484.s009.zip › 3D worm tracker code/sample/2_16.tif]

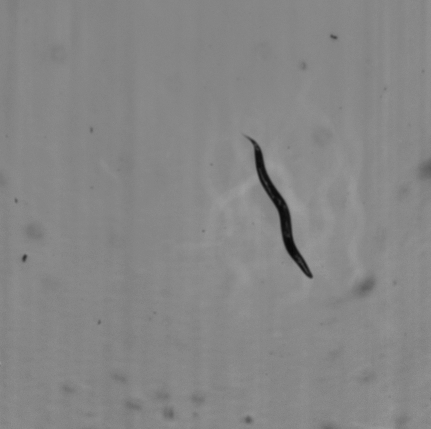

Supplement: Dataset S1 — MATLAB-based code for kinematic analysis of 3-D worm tracker. The *.zip file contains *.m files for reconstruction and visualization of a stereoscopic image set. It also contains sample images (30 frames with 1 sec intervals), and an CSV file including width at each region of a worm. The MATLAB based code requires MATLAB and Image Processing Toolbox. (ZIP) [file pone.0057484.s009.zip › 3D worm tracker code/sample/2_17.tif]

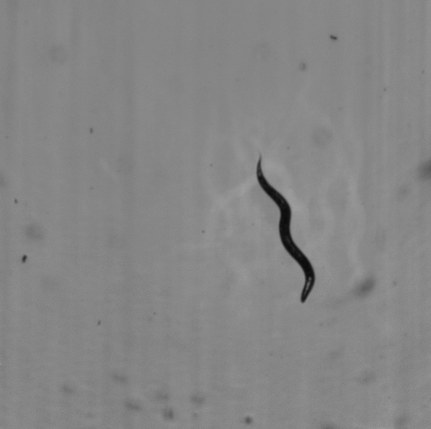

Supplement: Dataset S1 — MATLAB-based code for kinematic analysis of 3-D worm tracker. The *.zip file contains *.m files for reconstruction and visualization of a stereoscopic image set. It also contains sample images (30 frames with 1 sec intervals), and an CSV file including width at each region of a worm. The MATLAB based code requires MATLAB and Image Processing Toolbox. (ZIP) [file pone.0057484.s009.zip › 3D worm tracker code/sample/2_18.tif]

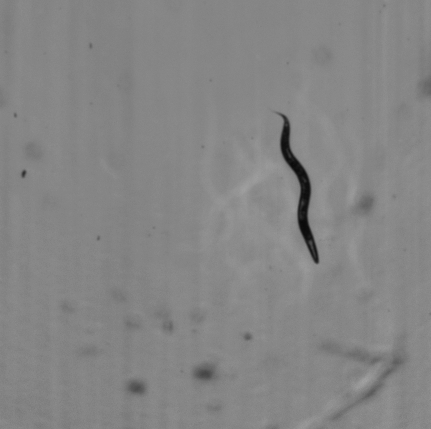

Supplement: Dataset S1 — MATLAB-based code for kinematic analysis of 3-D worm tracker. The *.zip file contains *.m files for reconstruction and visualization of a stereoscopic image set. It also contains sample images (30 frames with 1 sec intervals), and an CSV file including width at each region of a worm. The MATLAB based code requires MATLAB and Image Processing Toolbox. (ZIP) [file pone.0057484.s009.zip › 3D worm tracker code/sample/2_19.tif]

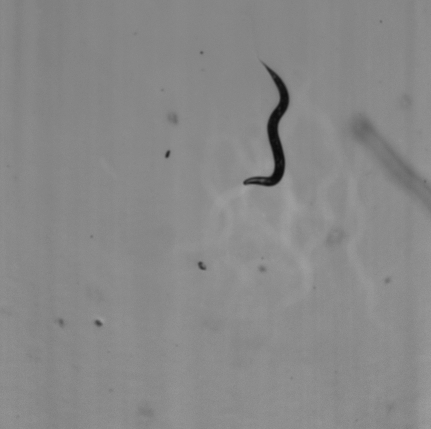

Supplement: Dataset S1 — MATLAB-based code for kinematic analysis of 3-D worm tracker. The *.zip file contains *.m files for reconstruction and visualization of a stereoscopic image set. It also contains sample images (30 frames with 1 sec intervals), and an CSV file including width at each region of a worm. The MATLAB based code requires MATLAB and Image Processing Toolbox. (ZIP) [file pone.0057484.s009.zip › 3D worm tracker code/sample/2_2.tif]

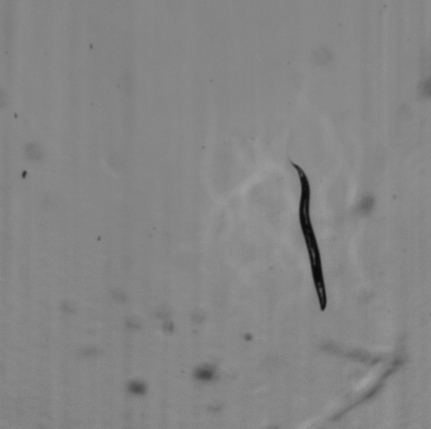

Supplement: Dataset S1 — MATLAB-based code for kinematic analysis of 3-D worm tracker. The *.zip file contains *.m files for reconstruction and visualization of a stereoscopic image set. It also contains sample images (30 frames with 1 sec intervals), and an CSV file including width at each region of a worm. The MATLAB based code requires MATLAB and Image Processing Toolbox. (ZIP) [file pone.0057484.s009.zip › 3D worm tracker code/sample/2_20.tif]

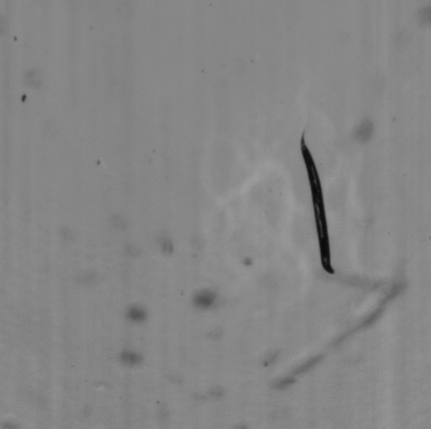

Supplement: Dataset S1 — MATLAB-based code for kinematic analysis of 3-D worm tracker. The *.zip file contains *.m files for reconstruction and visualization of a stereoscopic image set. It also contains sample images (30 frames with 1 sec intervals), and an CSV file including width at each region of a worm. The MATLAB based code requires MATLAB and Image Processing Toolbox. (ZIP) [file pone.0057484.s009.zip › 3D worm tracker code/sample/2_21.tif]

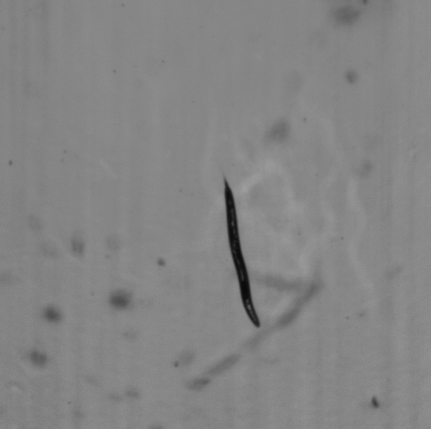

Supplement: Dataset S1 — MATLAB-based code for kinematic analysis of 3-D worm tracker. The *.zip file contains *.m files for reconstruction and visualization of a stereoscopic image set. It also contains sample images (30 frames with 1 sec intervals), and an CSV file including width at each region of a worm. The MATLAB based code requires MATLAB and Image Processing Toolbox. (ZIP) [file pone.0057484.s009.zip › 3D worm tracker code/sample/2_22.tif]

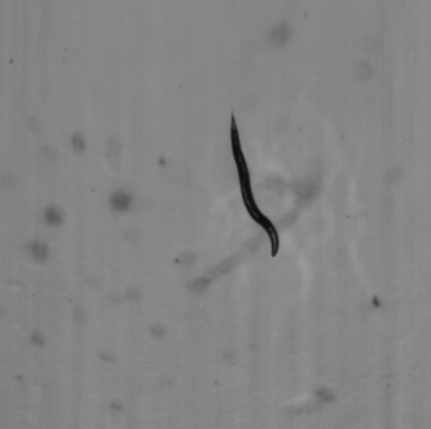

Supplement: Dataset S1 — MATLAB-based code for kinematic analysis of 3-D worm tracker. The *.zip file contains *.m files for reconstruction and visualization of a stereoscopic image set. It also contains sample images (30 frames with 1 sec intervals), and an CSV file including width at each region of a worm. The MATLAB based code requires MATLAB and Image Processing Toolbox. (ZIP) [file pone.0057484.s009.zip › 3D worm tracker code/sample/2_23.tif]

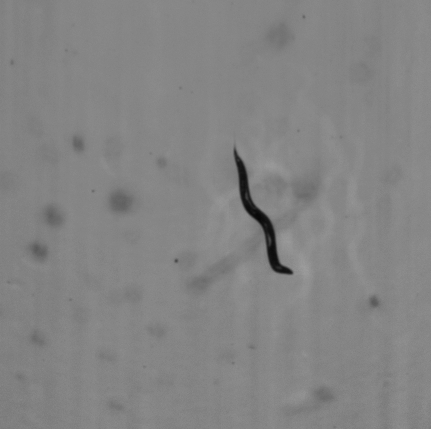

Supplement: Dataset S1 — MATLAB-based code for kinematic analysis of 3-D worm tracker. The *.zip file contains *.m files for reconstruction and visualization of a stereoscopic image set. It also contains sample images (30 frames with 1 sec intervals), and an CSV file including width at each region of a worm. The MATLAB based code requires MATLAB and Image Processing Toolbox. (ZIP) [file pone.0057484.s009.zip › 3D worm tracker code/sample/2_24.tif]

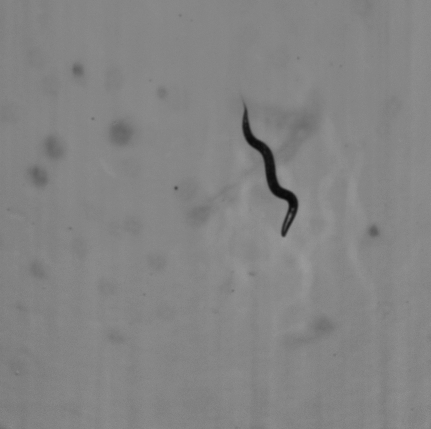

Supplement: Dataset S1 — MATLAB-based code for kinematic analysis of 3-D worm tracker. The *.zip file contains *.m files for reconstruction and visualization of a stereoscopic image set. It also contains sample images (30 frames with 1 sec intervals), and an CSV file including width at each region of a worm. The MATLAB based code requires MATLAB and Image Processing Toolbox. (ZIP) [file pone.0057484.s009.zip › 3D worm tracker code/sample/2_25.tif]

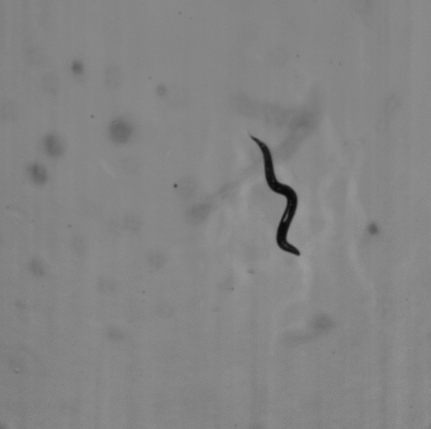

Supplement: Dataset S1 — MATLAB-based code for kinematic analysis of 3-D worm tracker. The *.zip file contains *.m files for reconstruction and visualization of a stereoscopic image set. It also contains sample images (30 frames with 1 sec intervals), and an CSV file including width at each region of a worm. The MATLAB based code requires MATLAB and Image Processing Toolbox. (ZIP) [file pone.0057484.s009.zip › 3D worm tracker code/sample/2_26.tif]

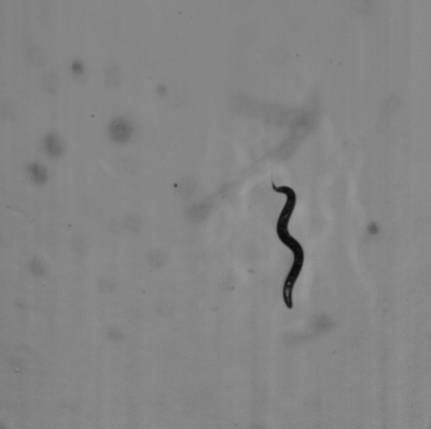

Supplement: Dataset S1 — MATLAB-based code for kinematic analysis of 3-D worm tracker. The *.zip file contains *.m files for reconstruction and visualization of a stereoscopic image set. It also contains sample images (30 frames with 1 sec intervals), and an CSV file including width at each region of a worm. The MATLAB based code requires MATLAB and Image Processing Toolbox. (ZIP) [file pone.0057484.s009.zip › 3D worm tracker code/sample/2_27.tif]

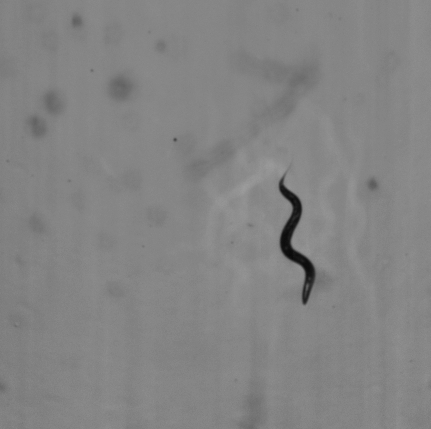

Supplement: Dataset S1 — MATLAB-based code for kinematic analysis of 3-D worm tracker. The *.zip file contains *.m files for reconstruction and visualization of a stereoscopic image set. It also contains sample images (30 frames with 1 sec intervals), and an CSV file including width at each region of a worm. The MATLAB based code requires MATLAB and Image Processing Toolbox. (ZIP) [file pone.0057484.s009.zip › 3D worm tracker code/sample/2_28.tif]

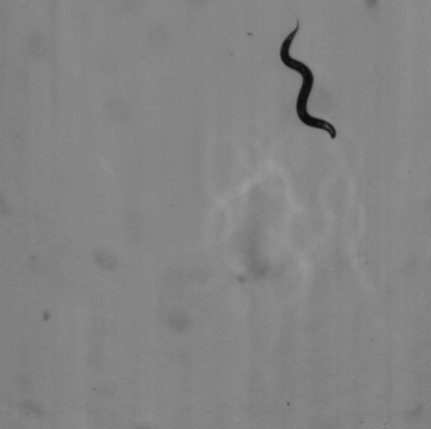

Supplement: Dataset S1 — MATLAB-based code for kinematic analysis of 3-D worm tracker. The *.zip file contains *.m files for reconstruction and visualization of a stereoscopic image set. It also contains sample images (30 frames with 1 sec intervals), and an CSV file including width at each region of a worm. The MATLAB based code requires MATLAB and Image Processing Toolbox. (ZIP) [file pone.0057484.s009.zip › 3D worm tracker code/sample/2_29.tif]

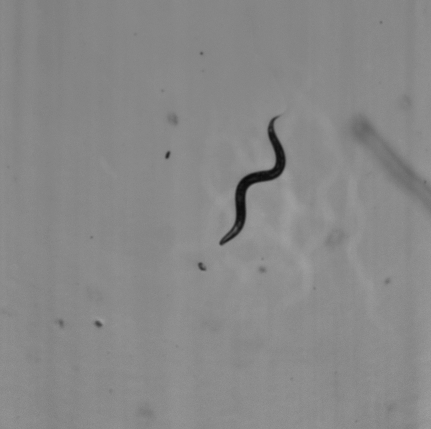

Supplement: Dataset S1 — MATLAB-based code for kinematic analysis of 3-D worm tracker. The *.zip file contains *.m files for reconstruction and visualization of a stereoscopic image set. It also contains sample images (30 frames with 1 sec intervals), and an CSV file including width at each region of a worm. The MATLAB based code requires MATLAB and Image Processing Toolbox. (ZIP) [file pone.0057484.s009.zip › 3D worm tracker code/sample/2_3.tif]

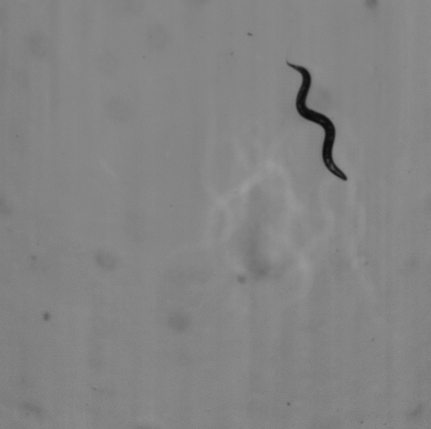

Supplement: Dataset S1 — MATLAB-based code for kinematic analysis of 3-D worm tracker. The *.zip file contains *.m files for reconstruction and visualization of a stereoscopic image set. It also contains sample images (30 frames with 1 sec intervals), and an CSV file including width at each region of a worm. The MATLAB based code requires MATLAB and Image Processing Toolbox. (ZIP) [file pone.0057484.s009.zip › 3D worm tracker code/sample/2_30.tif]

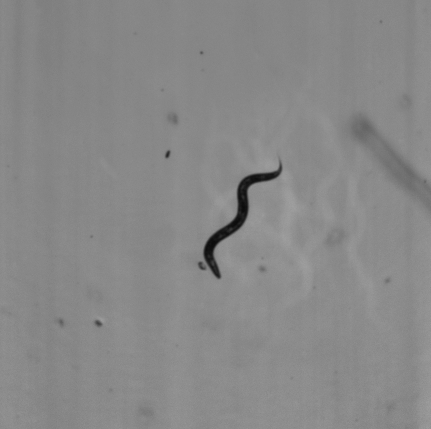

Supplement: Dataset S1 — MATLAB-based code for kinematic analysis of 3-D worm tracker. The *.zip file contains *.m files for reconstruction and visualization of a stereoscopic image set. It also contains sample images (30 frames with 1 sec intervals), and an CSV file including width at each region of a worm. The MATLAB based code requires MATLAB and Image Processing Toolbox. (ZIP) [file pone.0057484.s009.zip › 3D worm tracker code/sample/2_4.tif]

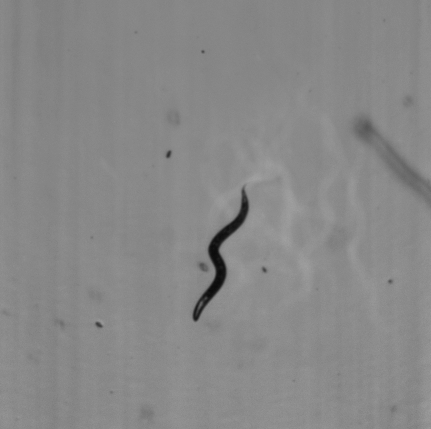

Supplement: Dataset S1 — MATLAB-based code for kinematic analysis of 3-D worm tracker. The *.zip file contains *.m files for reconstruction and visualization of a stereoscopic image set. It also contains sample images (30 frames with 1 sec intervals), and an CSV file including width at each region of a worm. The MATLAB based code requires MATLAB and Image Processing Toolbox. (ZIP) [file pone.0057484.s009.zip › 3D worm tracker code/sample/2_5.tif]

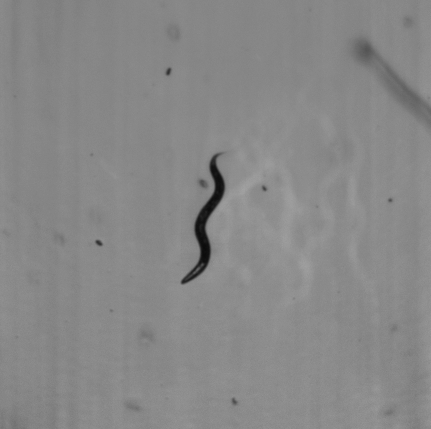

Supplement: Dataset S1 — MATLAB-based code for kinematic analysis of 3-D worm tracker. The *.zip file contains *.m files for reconstruction and visualization of a stereoscopic image set. It also contains sample images (30 frames with 1 sec intervals), and an CSV file including width at each region of a worm. The MATLAB based code requires MATLAB and Image Processing Toolbox. (ZIP) [file pone.0057484.s009.zip › 3D worm tracker code/sample/2_6.tif]

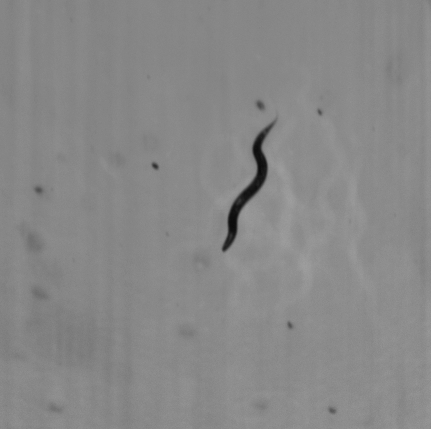

Supplement: Dataset S1 — MATLAB-based code for kinematic analysis of 3-D worm tracker. The *.zip file contains *.m files for reconstruction and visualization of a stereoscopic image set. It also contains sample images (30 frames with 1 sec intervals), and an CSV file including width at each region of a worm. The MATLAB based code requires MATLAB and Image Processing Toolbox. (ZIP) [file pone.0057484.s009.zip › 3D worm tracker code/sample/2_7.tif]

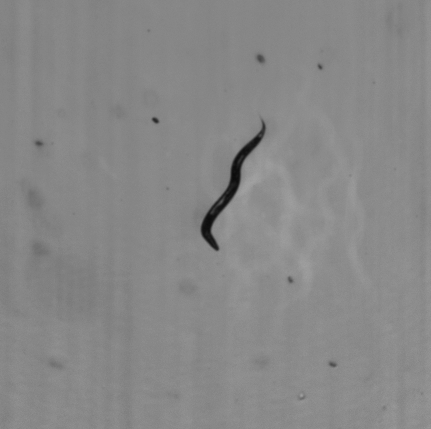

Supplement: Dataset S1 — MATLAB-based code for kinematic analysis of 3-D worm tracker. The *.zip file contains *.m files for reconstruction and visualization of a stereoscopic image set. It also contains sample images (30 frames with 1 sec intervals), and an CSV file including width at each region of a worm. The MATLAB based code requires MATLAB and Image Processing Toolbox. (ZIP) [file pone.0057484.s009.zip › 3D worm tracker code/sample/2_8.tif]

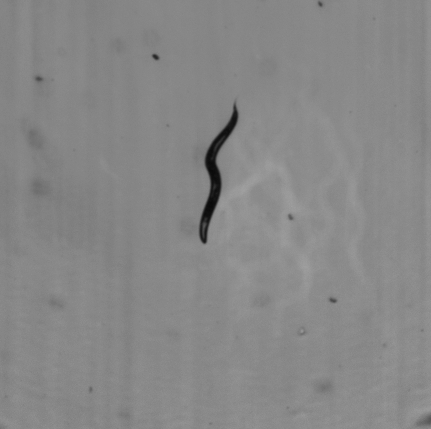

Supplement: Dataset S1 — MATLAB-based code for kinematic analysis of 3-D worm tracker. The *.zip file contains *.m files for reconstruction and visualization of a stereoscopic image set. It also contains sample images (30 frames with 1 sec intervals), and an CSV file including width at each region of a worm. The MATLAB based code requires MATLAB and Image Processing Toolbox. (ZIP) [file pone.0057484.s009.zip › 3D worm tracker code/sample/2_9.tif]
